# Risk Factors for the Onset of Frozen Shoulder in Middle-Aged and Elderly Subjects Within 1 Year of Discharge From a Hospitalization That Involved Intravenous Infusion: A Prospective Cohort Study

**DOI:** 10.3389/fmed.2022.911532

**Published:** 2022-06-20

**Authors:** Wenping Cao, Jiangnan Chen, Jianfeng Pu, Yunwu Fan, Ye Cao

**Affiliations:** ^1^Department of Acupuncture, Zhangjiagang Second People's Hospital, Zhangjiagang City, China; ^2^Department of Pain Medicine, Zhangjiagang Second People's Hospital, Zhangjiagang City, China

**Keywords:** frozen shoulder, hospitalized patients, intravenous fluid, risk factors, cohort study

## Abstract

**Aim:**

To investigate the incidence of frozen shoulder and risk factors for the onset of frozen shoulder in middle-aged and elderly subjects within 1 year of discharge from a hospitalization that involved intravenous infusion in Zhangjiagang Second People's Hospital.

**Methods:**

A total of 1,900 subjects who were discharged from a hospitalization that involved intravenous infusion in the hospital between May 2020 and September 2020 met the inclusion criteria for this study: 950 subjects had a mean daily duration of intravenous infusion ≤ 2 h (low exposure) and 950 subjects had a mean daily duration of intravenous infusion ≥3 h (high exposure). Subjects were followed up by telephone at 6 months ± 1 week and 12 months ± 1 week after discharge the incidence of frozen shoulder.

**Results:**

The cumulative incidence rate of frozen shoulder within 1 year of discharge was 5.2%. Multivariate logistic regression analysis revealed the risk of frozen shoulder was higher in subjects with a mean daily duration of intravenous infusion ≥3 h compared to ≤ 2 h (OR = 3.082, 95% CI 1.919–4.949, *P* < 0.001); subjects hospitalized for 11–30 days had a higher risk of frozen shoulder compared to those hospitalized for 10 days or less (OR = 6.836, 95%CI 4.363–10.709, *P* < 0.001); subjects who were overweight/ obese (BMI ≥ 25 kg/m^2^) had a higher risk of frozen shoulder compared to those of normal weight (BMI 18.5–24.9 kg/m^2^) (OR = 2.166, 95%CI 1.376–3.410, *P* = 0.001); subjects in the 56–70-year-old age group had a higher risk of developing frozen shoulder compared to those in the 40–55-year-old age group (OR = 1.977, 95%CI 1.154–3.387, *P* = 0.013); diabetes increased the risk of frozen shoulder (OR = 3.009, 95%CI 1.826–4.959, *P* < 0.001). The 71–85 years old age group and hypertension were statistically significant in univariate analysis but not in multivariate analysis (*P* > 0.05).

**Conclusion:**

Compared with middle-aged and elderly in the general population, middle-aged and elderly subjects who received intravenous infusion during a hospitalization had a higher cumulative incidence rate of frozen shoulder within 1 year after discharge. Independent risk factors for the onset of frozen shoulder included mean daily duration of intravenous infusion ≥3 h, length of hospital stay 11–30 days, BMI ≥ 25 kg/m^2^, age 56–70 years, and diabetes.

## Introduction

Frozen shoulder is a common clinical disorder. In addition to pain, frozen shoulder can cause a gradual reduction in the range of motion of the shoulder joint, which seriously impacts all areas of an affected individual's work and life. Globally, the incidence of frozen shoulder is estimated at 2–5% (1). In 1934, Codman introduced the term “frozen shoulder” (2). In 1945, Neviaser named the condition “adhesive capsulitis” (3). In 2011, the American Society of Shoulder and Elbow Surgeons (ASES) (4) defined frozen shoulder as: “a morbid state of the shoulder joint in which both active and passive mobility of the shoulder joint are limited. Except for the possibility of osteopenia or calcific tendinitis, there is no obvious change in the glenohumeral joint on X-ray.”

Frozen shoulder is a self-limiting condition. The course of disease is generally between 2 and 3 years, but 50–70% of patients will have varying degrees of shoulder pain and limited mobility for a longer period of time (5, 6). Common interventions for frozen shoulder include physical therapy, acupuncture, manipulation, orally administered medications, local steroid injection, manual release under anesthesia, and arthroscopic capsular release (7). These approaches may be successful in the short-term; however, symptoms such as shoulder pain and limited mobility gradually worsen with time (1, 8). There is no cure for frozen shoulder, as there is no recognized effective treatment; therefore, research should focus on identifying risk factors for frozen shoulder, which will broaden knowledge about prevention and treatment and reduce the incidence of frozen shoulder.

The etiology of the frozen shoulder has not been fully understood. The Upper Limb Committee of the International Society of Arthroscopy, Knee Surgery and Orthopedic Sports Medicine (ISAKOS) classify frozen shoulder as primary (idiopathic) or secondary (shoulder trauma [fracture, dislocation, and soft tissue injury]; non-traumatic osteoarthritis, rotator cuff injury, calcific tendinitis, prolonged immobilization of the shoulder joint after surgery, injury to the cervical spine or brachial plexus) (6, 9, 10). Risk factors for primary frozen shoulder include diabetes, Dupuytren's contracture, thyroid disease, myocardial infarction, and Parkinson's disease (1). In other studies, the incidence of frozen shoulder has been associated with occupational factors, whereby individuals working at high altitudes, handling heavy objects and performing manual labor are more likely to suffer from frozen shoulder (11).

In our acupuncture department, we treat a large number of patients with frozen shoulder each year. While obtaining routine medical history from our patients, we found that some patients had been hospitalized and received intravenous infusion prior to experience frozen shoulder, and they had shoulder pain and limited mobility for some time after discharge without obvious predisposing factors. A previous report identified prolonged post-operative intravenous infusion as a risk factor for frozen shoulder in patients who underwent neurosurgery (12). Previously, in a preliminary study that included small sample size, we found that the duration of intravenous infusion in inpatients was associated with the incidence of frozen shoulder. The objective of the present study was to investigate the incidence of frozen shoulder and risk factors for the onset of frozen shoulder in middle-aged and elderly subjects within 1 year of discharge from a hospitalization that involved intravenous infusion in the Zhangjiagang Second People's Hospital. Findings should contribute to the development of strategies to prevent the onset of frozen shoulder after hospitalization with intravenous infusion.

## Materials and Methods

### Study Design

This single-center, prospective cohort study was conducted in compliance with the Declaration of Helsinki (13). The research protocol was reviewed by the Ethics Committee of Zhangjiagang Second People's Hospital (approval number: ZEY−2020007), and was registered in the Chinese Clinical Trial Registry, ChiCTR (http://www.chictr.org.cn; registration number: ChiCTR2000031862). In this study, the exposure was intravenous infusion, which uses the principles of atmospheric pressure and hydrostatic pressure to infuse a large amount of sterile fluid, electrolytes, and/or drugs into the body. The outcome was incidence of frozen shoulder. The mean daily duration of intravenous infusion was calculated as total duration of intravenous infusion during hospitalization divided by the number of hospitalization days. According to our previous unpublished study, subjects were stratified based on mean daily duration of intravenous infusion as high exposure (mean daily duration of intravenous infusion ≥3 h) or low exposure (mean daily duration of intravenous infusion ≤ 2 h).

### Sample Size Calculation

PASS 11 software was used to estimate the sample size according to the relative risk (RR) of the outcome of the two groups of subjects that underwent intravenous infusion. We conducted a preliminary study in middle-aged and elderly subjects who were discharged from a hospitalization that involved intravenous infusion. Findings showed that the incidence of frozen shoulder was 6.7% in the high-exposure group and 3.3% in the low-exposure group, with an RR of 2.03. The present study included an equal number of subjects in the low and high exposure groups. With α = 0.05 and power 1-β = 0.9, the effective sample size for each group was calculated as *n* = 862. Based on clinical experience, we expected a 10% drop-out rate; therefore, the effective sample size for each group was calculated as *n* = 950.

### Study Subjects

Subjects who were discharged from a hospitalization that involved intravenous infusion in the Second People's Hospital of Zhangjiagang City, Jiangsu Province, China between May 2020 and September 2020 were eligible for this study. Inclusion criteria were: (1) subjects discharged from a hospitalization that involved intravenous infusion; (2) subjects aged 40–85 years; (3) subjects hospitalized for 1–30 days; (4) subjects signed an informed consent form upon discharge and agreed to post-discharge follow up. Exclusion criteria were: (1) subjects aged <40 years or >85 years; (2) subjects hospitalized for >30 days; (3) subjects admitted to hospital due to trauma-induced shoulder pain and dysfunction, such as shoulder sprain, fracture, dislocation, rupture of the supraspinatus tendon, etc. (4) subjects who had frozen shoulder before admission and had recovered, or had frozen shoulder at admission and during hospitalization; (5) subjects who had undergone craniocerebral or other neurosurgical procedures before admission or during hospitalization, or suffered from intracranial lesions etc., and had poor recovery after discharge, resulting in decreased muscle strength of the neck and shoulder (muscle strength ≤ grade 4), causing limited activity of the shoulder joint; (6) subjects who underwent surgery on the shoulder, neck, chest, and other parts that would later lead to stiffness and adhesion of the soft tissue around the shoulder joint; (7) subjects with mental disorders, such as dementia, psychiatric diagnosis, and intellectual retardation etc, or who could not clearly express themselves for other reasons; (8) subjects who were unwilling to sign the informed consent form or expressly stated that they would not comply with follow-up.

### Outcome Measure

The primary endpoint was the incidence of frozen shoulder within 1 year of discharge from a hospitalization that involved intravenous infusion. The secondary endpoint was the identification of risk factors for the onset of frozen shoulder following discharge from a hospitalization that involved intravenous infusion.

### Data Collection

We included subjects in order of discharge until we met the required sample size for each group. During the period we included a total of 1,900 subjects meeting the criteria. There were 950 cases in each group of exposure group and control group. The hospital records of included subjects were reviewed. Demographic characteristics (gender, age, body mass index [BMI], education, work status) and clinical characteristics (diabetes, hypertension, surgery, length of stay, mean daily duration of intravenous infusion) were recorded.

Follow-up was calculated from the day of hospital discharge. Subjects were followed-up by telephone at 6 months ± 1 week and 12 months ± 1 week after hospital discharge by specially trained staff. Subjects were asked whether they had shoulder pain and limited mobility. Subjects who reported shoulder pain and limitation of activities were asked whether there were any predisposing factors such as shoulder joint trauma or central and peripheral nerve injury. Subjects with no predisposing factors were instructed to attend the hospital for a follow-up visit, where frozen shoulder was diagnosed based on criteria reported in the literature (9, 14, 15). If a subject clearly stated that there were no predisposing factors and they refused to come to the hospital for examination, shoulder pain, and limited activity were considered unrelated to frozen shoulder, and follow-up ended.

At the follow-up hospital visit, an experienced orthopedic surgeon asked the subjects about their medical history and conducted a physical examination. X-ray, MRI, or other examinations may have been performed to exclude other joint and soft tissue disorders such as acromioclavicular arthritis, rheumatoid arthritis, septic arthritis, supraspinatus tendonitis, subacromial bursitis or biceps long head tendonitis; bone structural abnormalities such as osteonecrosis, primary and metastatic tumors or Paget's disease; neck lesions such as cervical spondylosis or thoracic outlet syndrome; or shoulder pain caused by visceral lesions such as upper lung tumor, esophagitis, myocardial infarction, digestive tract ulcers, or cholecystitis. This procedure screened out subjects who did not meet the diagnostic criteria for frozen shoulder, those with frozen shoulder were included in the analysis.

### Statistical Analysis

Statistical analysis was performed using SPSS 21.0 software. Multiple imputation was used to handle missing data (16). Categorical variables were reported as number of cases (%) and compared with the χ^2^ test. Rate difference and corresponding 95% confidence intervals (95% CIs) were calculated with the Wilson method. The associations between relevant risk factors and the onset of frozen shoulder were explored with binary logistic regression analysis. The dependent variable, frozen shoulder, was binary (not occur = 0; occur = 1), and the independent variables were binary or ordered multi-category. Binary variables were: mean daily duration of intravenous infusion (≤ 2 h = 0; ≥3 h = 1), gender (male = 0; female = 1), work status (no = 0; yes = 1), hypertension (no = 0; yes = 1), diabetes (no = 0; yes = 1), surgical history (no = 0; yes = 1), and length of hospital stay (≤ 10 days = 0, 11–30 days = 1). Ordered multi-category variables were: age (40–55 years = 1; 56–70 years = 2; 71–85 years = 3), BMI (18.5–24.9 kg/m^2^ = 1; <18.5 kg/m^2^ = 2; ≥25 kg/m^2^ = 3), and education (primary school and below = 1; middle school = 2; university and above = 3). Potential risk factors were identified with univariate analysis. Factors with *P* < 0.05 were recruited into multivariate logistic regression analysis. Odds ratios (OR) with 95% CIs were calculated. *P* < 0.05 was considered statistically significant.

## Results

### Study Subjects

**Figure 1:**
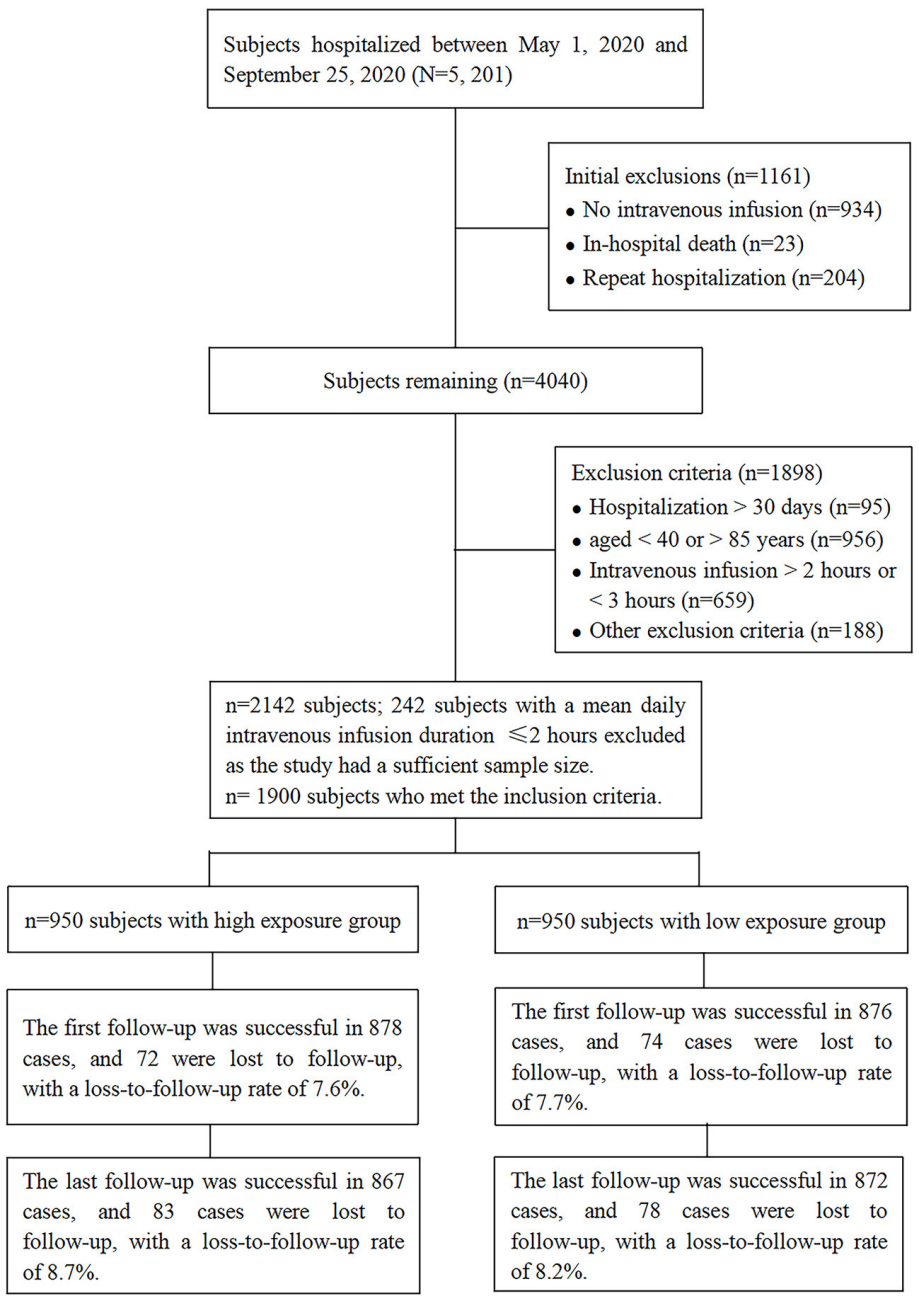
Study flow chart.

**Figure 2:**
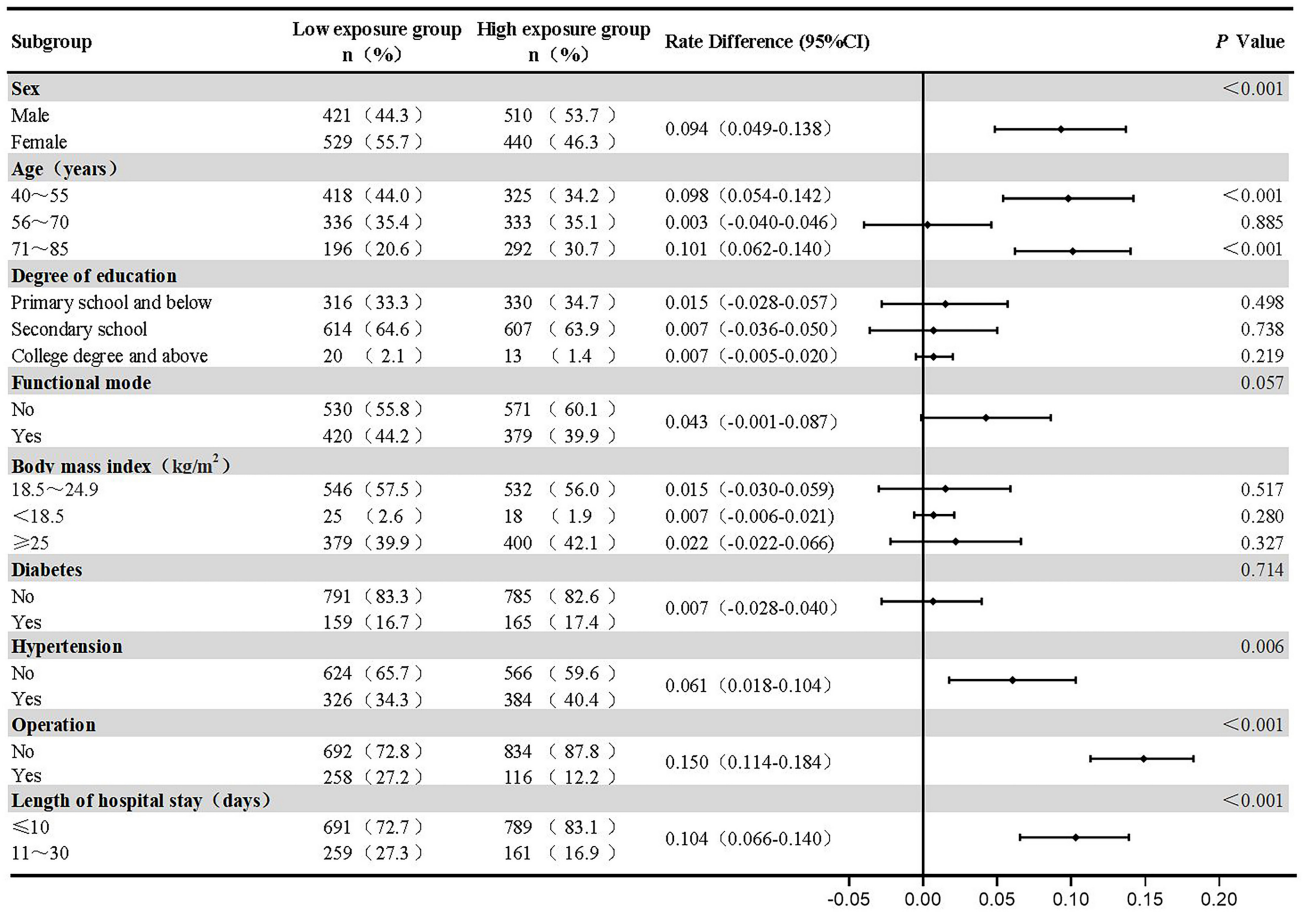
Subjects' baseline demographic and clinical characteristics stratified by mean daily duration of intravenous infusion.

A total of 5,201 consecutive subjects who were discharged from the Second People's Hospital of Zhangjiagang City, Jiangsu Province, China between May 1, 2020 and September 25, 2020 were screened to determine their eligibility for inclusion in this study. Among these, 4,267 subjects received an intravenous infusion, and 2,125 patients were excluded as they met the study's predefined exclusion criteria. Subjects were recruited until the required sample size for each group was met. Among eligible subjects, 242 subjects were excluded after the sample size for the low exposure group was met. Finally, the study included 1,900 subjects: 950 subjects with a mean daily duration of intravenous infusion ≤ 2 h (low exposure) and 950 subjects with a mean daily duration of intravenous infusion ≥3 h (high exposure). Overall, 78 (8.2%) subjects with a mean daily duration of intravenous infusion ≤ 2 h (low exposure) and 83 (8.7%) subjects with a mean daily duration of intravenous infusion ≥3 h (high exposure) were lost to follow-up, with no significant difference in lost to follow-up rates between subjects with low and high exposure to intravenous infusion (RD = 0.005, 95% CI −0.020–0.031, *P* = 0.680) (Figure 1). Subjects' baseline demographic and clinical characteristics stratified by mean daily duration of intravenous infusion are summarized in Figure 2.

### Incidence of Frozen Shoulder

During follow-up, a total of 98 subjects had frozen shoulders, for a cumulative incidence rate of 5.2%: 31 subjects with a mean daily duration of intravenous infusion ≤ 2 h were diagnosed with frozen shoulder, for a cumulative incidence rate of 3.3%; 67 subjects with a mean daily duration of intravenous infusion ≥3 h were diagnosed with frozen shoulder, for a cumulative incidence rate of 7.1% (RR = 2.15).

At the first follow-up, 79 subjects reported shoulder pain (*n* = 29, mean daily duration of intravenous infusion ≤ 2 h; *n* = 50 mean daily duration of intravenous infusion ≥3 h), and 65 subjects were diagnosed with frozen shoulder (*n* = 20, mean daily duration of intravenous infusion ≤ 2 h; *n* = 45 mean daily duration of intravenous infusion ≥3 h), for an incidence rate of 3.4%, and accounting for 66.3% of the cumulative cases.

At the last follow-up, 64 subjects reported shoulder pain (*n* = 23, mean daily duration of intravenous infusion ≤ 2 h; *n* = 41 mean daily duration of intravenous infusion ≥3 h), and 33 subjects were diagnosed with frozen shoulder (*n* = 11, mean daily duration of intravenous infusion ≤ 2 h; *n* = 22 mean daily duration of intravenous infusion ≥3 h), accounting for 33.7% of the cumulative cases.

### Univariate Analysis

**Figure 3:**
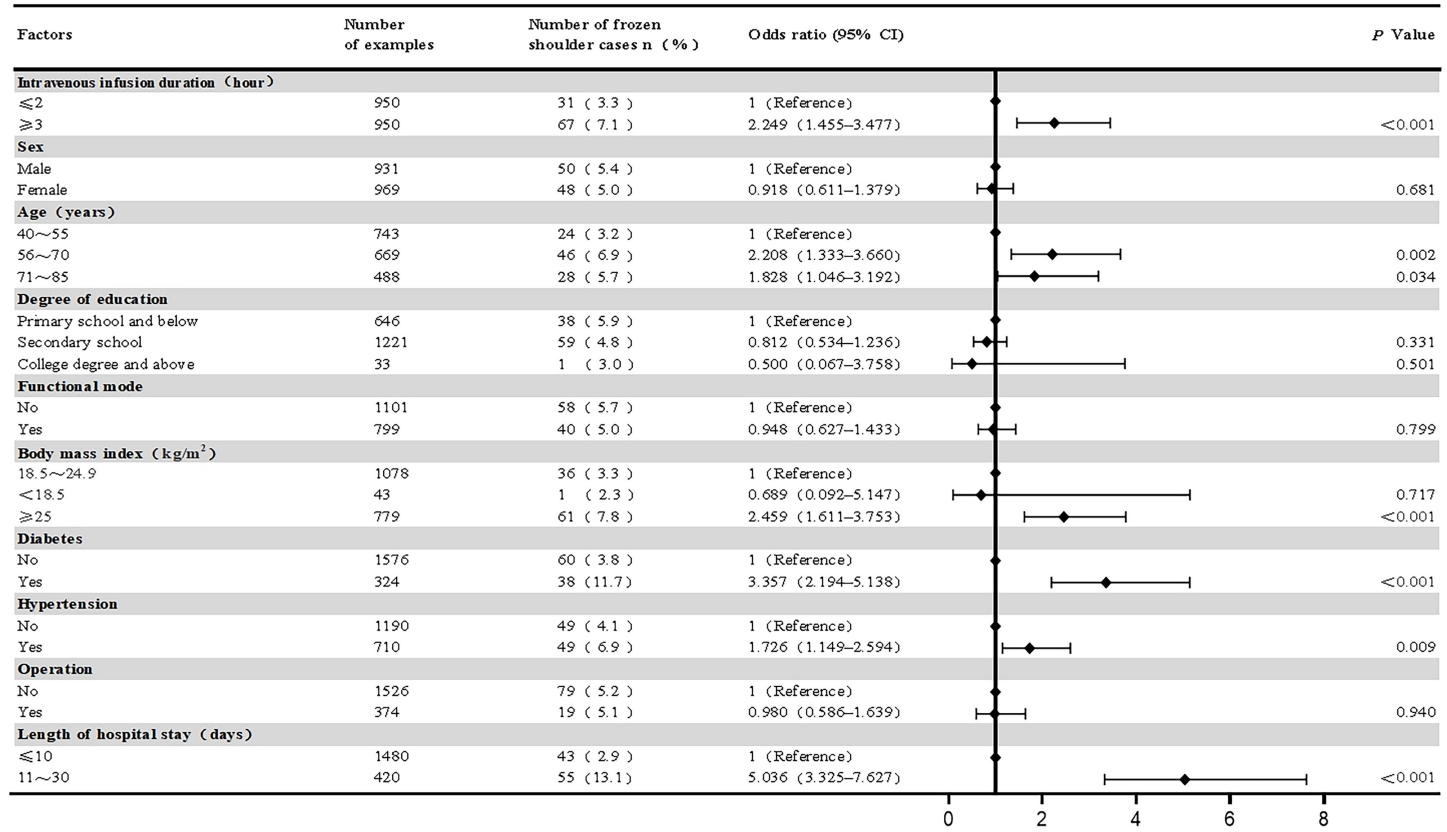
Univariate binary logistic regression analysis on the risk of frozen shoulder in study subjects.

Univariate analysis revealed significant associations between mean daily duration of intravenous infusion, length of hospital stay, BMI, age, diabetes, and hypertension and the onset of frozen shoulder (*P* < 0.05). There were no significant associations between subjects' gender, education, work status, and surgery and the onset of frozen shoulder (Figure 3).

### Multivariate Analysis

Multivariate analysis revealed longer mean daily duration of intravenous infusion, longer length of hospital stay, BMI ≥ 25 kg/m^2^, age 56–70 years, and diabetes were independent risk factors for the onset of frozen shoulder in middle-aged and elderly subjects within 1 year of discharge from a hospitalization that involved intravenous infusion.

The Hosmer-Lemeshow goodness of fit index for the multivariate logistic regression model was good (*P* = 0.426). The model could correctly classify 95.2% of the predicted values. The model sensitivity was 6.1%, specificity was 100%, positive predictive value was 100%, and negative predictive value was 95.1%.

**Figure 4:**
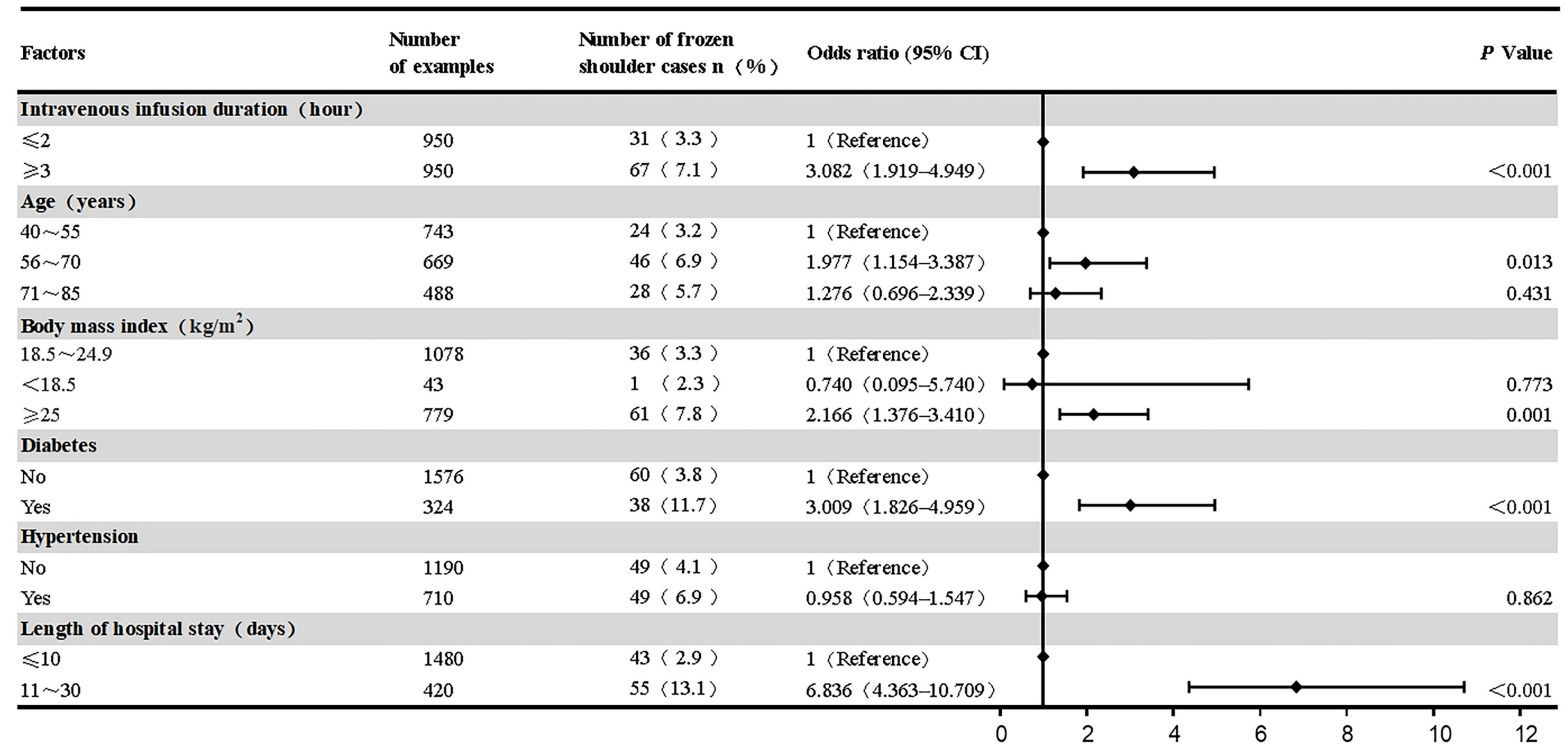
Multivariate binary logistic regression analysis on the risk of frozen shoulder in study subjects.

Multivariate logistic regression analysis revealed the risk of frozen shoulder was higher in subjects with a mean daily duration of intravenous infusion ≥3 h compared to ≤ 2 h (OR = 3.082, 95% CI 1.919–4.949, *P* < 0.001); subjects hospitalized for 11–30 days had a higher risk of frozen shoulder compared to those hospitalized for 10 days or less (OR = 6.836, 95%CI 4.363–10.709, *P* < 0.001); subjects who were overweight/ obese (BMI ≥ 25 kg/m^2^) had a higher risk of frozen shoulder compared to those of normal weight (BMI 18.5–24.9 kg/m^2^) (OR = 2.166, 95%CI 1.376–3.410, *P* = 0.001); subjects in the 56–70-year-old age group had a higher risk of developing frozen shoulder compared to those in the 40–55-year-old age group (OR = 1.977, 95% CI 1.154–3.387, *P* = 0.013); diabetes increased the risk of frozen shoulder (OR = 3.009, 95% CI 1.826–4.959, *P* < 0.001). The 71–85 years old age group and hypertension were statistically significant in univariate analysis but not in multivariate analysis (*P* > 0.05) (Figure 4).

## Discussion

This study investigated the incidence of frozen shoulder and risk factors for the onset of frozen shoulder in middle-aged and elderly subjects within 1 year of discharge from a hospitalization that involved intravenous infusion. Findings showed the cumulative incidence rate of frozen shoulder within 1 year of discharge was 5.2%, with frozen shoulder within 6 months of discharge accounting for 66.3% of the cumulative cases. Independent risk factors for the onset of frozen shoulder in middle-aged and elderly subjects who were discharged from a hospitalization that involved intravenous infusion included mean daily duration of intravenous infusion ≥3 h, length of hospital stay 11–30 days, BMI ≥ 25 kg/m^2^, age 56–70 years, and diabetes.

The incidence of frozen shoulder among the middle-aged and elderly subjects included in this study was higher than previously reported for the general population. In Europe and the US, frozen shoulder affects an estimated 2% of the general population, with a cumulative incidence of 2.4 cases per 1,000 person-years (17); in the UK, the annual incidence of frozen shoulder in the general population is ~1.4 per 1,000 individuals (18); and in the US, the 1-year prevalence of frozen shoulder in individuals aged >65 years is 0.35% (19). In China, intravenous infusion is the most common mode of administration of medications, nutrients and fluids in inpatients; in 2016, 93.1% of inpatients in urban hospitals in China received intravenous medication administration (20). Complications associated with establishing an intravenous route for administering therapy (infiltration, hematoma, air embolism, phlebitis, extravascular drug administration, intraarterial injection) have been well-documented (21); however, reports on the long-term sequelae of intravenous infusion are scarce.

In the present study, mean daily duration of intravenous infusion and length of hospital stay were independent risk factors for the onset of frozen shoulder in middle-aged and elderly subjects within 1 year of discharge from a hospitalization that involved intravenous infusion. Patients must temporarily limit upper extremity activities during intravenous infusion. A long duration of intravenous infusion and prolonged hospital stay will cause substantial limitations on upper extremity activities, increasing the incidence of frozen shoulder. A study in patients who had surgical treatment for sub-arachnoid hemorrhage reported the incidence of frozen shoulder at 6 months of follow-up was 25.3%, and the development of frozen shoulder was associated with duration of post-operative intravenous infusion (12). Consistent with this, patients undergoing breast surgery and elderly patients with predisposed joint disease develop frozen shoulder following long periods of immobilization (22). The ISAKOS Upper Limb Committee proposed that joint capsule contracture after long-term immobilization of the shoulder joint and muscle tension around the shoulder joint can cause frozen shoulder (9).

Accumulating evidence suggests that diabetes and hypertension are risk factors for the onset of frozen shoulder (1, 23, 24). Specifically, frozen shoulder occurs 2–5 times more frequently in individuals with diabetes compared to those without (25, 26). In the present study, middle-aged and elderly subjects with diabetes had an increased risk of developing frozen shoulder within 1 year of discharge from a hospitalization that involved intravenous infusion, with an OR of 3.009. There was a significant association between hypertension and the onset of frozen shoulder on univariate analysis (*P* = 0.009); however, hypertension was not an independent risk factor for frozen shoulder on multivariate analysis (*P* = 0.862). Evidence suggests that the incidence of hypertension increases with age and in subjects with diabetes, implying an interaction between hypertension and age and diabetes. Accordingly, we performed *ad-hoc* analyses. Consistency testing of our data using SPSS 21.0 software showed that the OR values for hypertension and frozen shoulder in subjects aged 40–55, 56–70, and 71–85 years, were 1.775, 1.711, and 1.144, and the incidence of frozen shoulder tended to decrease with increasing age, indicating that age was an effect modifier for hypertension. The OR values for hypertension and frozen shoulder in subjects with no diabetes or diabetes were 1.445 and 0.856, respectively, which indicated that the pathogenesis of hypertension on frozen shoulder was affected by diabetes.

Some studies showed no association between obesity and the incidence of frozen shoulder (27, 28). Other reports suggested that the incidence of frozen shoulder was higher in obese people (23, 26), with one report showing obesity was a risk factor for frozen shoulder in individuals in Shanghai, China (29). Our study implied that overweight and obese subjects were more likely to develop frozen shoulder than subjects of normal-weight.

Many studies (17, 30) have shown that frozen shoulder is more common in individuals aged between 40 and 70 years (31, 32). To maximize the number of cases of frozen shoulder, and match the number of independent variables in this study, we included middle-aged and elderly subjects aged 40–85 years. Brun et al. (33) indicated that the peak age of onset of frozen shoulder was 56 years old. Saito et al. (32) reported that the mean age of onset of frozen shoulder was 58 years. In the present study, subjects aged 56–70 years were most likely to develop frozen shoulder within 1 year of discharge from a hospitalization that involved intravenous infusion.

Previous studies showed frozen shoulder is more common in women than men, with women comprising an estimated 58.0–60.9% of subjects with frozen shoulder (34, 35). Rawat et al. (36) found 68.75% of subjects with frozen shoulder were women. In the present study, there was no significant difference in the proportion of males and females suffering from frozen shoulder (RD = 0.004, 95%CI −0.016–0.025, *P* = 0.681). This may be because our study was limited to a specific population of subjects that received intravenous infusion during a hospitalization, gender-specific differences in the onset of frozen shoulder have been influenced by the effect of intravenous infusion or other confounders.

## Study Limitations

(1) This study only analyzed subjects receiving inpatient intravenous infusion in a single center and the study sample was limited by conditions, which affected the extrapolation of the study results. (2) The sample size was small and the incidence of frozen shoulder in the study population was low, resulting in a relatively small number of total cases. (3) The baseline characteristics of subjects with a mean daily duration of intravenous infusion ≤ 2 h (low exposure) and a mean daily duration of intravenous infusion ≥3 h (high exposure) were not matched using propensity scores; therefore, our findings may have been influenced by cofounders. (4) Our analyses did not consider mean daily durations of intravenous infusion between 2 and 3 h.

## Conclusion

Compared with middle-aged and elderly subjects in the general population, middle-aged, and elderly subjects who received intravenous infusion during a hospitalization had a higher cumulative incidence rate of frozen shoulder within 1 year after discharge, and most incidences of frozen shoulder occurred within 6 months after discharge. Risk factors for the onset of frozen shoulder in middle-aged and elderly subjects discharged from a hospitalization that involved intravenous infusion were mean daily duration of intravenous infusion ≥3 h, length of hospital stay 11–30 days, BMI ≥ 25 kg/m^2^, age 56–70 years, and diabetes. In middle-aged and elderly subjects that undergo intravenous infusion during hospitalization, the incidence of frozen shoulder after discharge may be reduced by accurate identification of these risk factors and timely intervention with appropriate functional exercises.

## Data Availability Statement

The datasets presented in this study can be found in online repositories. The names of the repository/repositories and accession number(s) can be found below: Clinical trial public platform management (http://www.medresman.org.cn).

## Ethics Statement

The studies involving human participants were reviewed and approved by Ethics Committee of Zhangjiagang Second People's Hospital. The patients/participants provided their written informed consent to participate in this study.

## Author Contributions

YC is the guarantor of this work and, as such, had full access to all the data in the study and takes responsibility for the integrity of the data and the accuracy of the data analysis. WC conceived and designed the study and wrote the manuscript. JC performed the statistical analysis. JP and YF carried out the literature search and data collection. WC and JC conducted an investigation. YC and JC revised the manuscript. All authors contributed to the article and approved the submitted version.

## Funding

This research was financed by science and technology support plan of Zhangjiagang City in 2020 [Grant No. ZKS2027] and Zhangjiagang City Health Youth Science and Technology Projects in 2020 [Grant No. ZJGQNKJ202037].

## Conflict of Interest

The authors declare that the research was conducted in the absence of any commercial or financial relationships that could be construed as a potential conflict of interest.

## Publisher's Note

All claims expressed in this article are solely those of the authors and do not necessarily represent those of their affiliated organizations, or those of the publisher, the editors and the reviewers. Any product that may be evaluated in this article, or claim that may be made by its manufacturer, is not guaranteed or endorsed by the publisher.
